# The Efficacy and Safety of Albumin‐Bound Paclitaxel Combined With Anlotinib and PD‐1/L1 Inhibitors For Treating Patients With Extensive‐Stage Small Cell Lung Cancer and Brain Metastasis: A Retrospective Cohort Study

**DOI:** 10.1002/cam4.70449

**Published:** 2024-12-11

**Authors:** Xiaobing Li, De Wu, Yi Peng, Jing Tang, Yuebing Wu

**Affiliations:** ^1^ Department of Thoracic Oncology, Hubei Cancer Hospital, Tongji Medical College Huazhong University of Science and Technology Wuhan China; ^2^ Department of Pathology, Hubei Cancer Hospital, Tongji Medical College Huazhong University of Science and Technology Wuhan China; ^3^ Department of Radiotherapy, Hubei Cancer Hospital, Tongji Medical College Huazhong University of Science and Technology Wuhan China; ^4^ Department of Lymphoma, Hubei Cancer Hospital, Tongji Medical College Huazhong University of Science and Technology Wuhan China

**Keywords:** albumin‐bound paclitaxel, anlotinib, brain metastasis, PD‐1, SCLC

## Abstract

**Objectives:**

Extensive‐stage small cell lung cancer (ES‐SCLC) suffering from brain metastases (BM) has a poor prognosis and lacks effective treatment selection. In this study, we explored the efficacy and safety of combination treatment of albumin‐bound paclitaxel (nab‐ptx), anlotinib, and PD‐1/L1 inhibitors for such special population.

**Methods:**

A total of 55 patients diagnosed with ES‐SCLC and BM were enrolled in this retrospective study. Patients received a combination therapy consisting of nab‐ptx, anlotinib, and PD‐1/L1 inhibitors. The primary endpoints included overall response rate (ORR), progression‐free survival (PFS), overall survival (OS), and adverse events (AEs).

**Results:**

The results demonstrated promising efficacy of the combination therapy for such patients, with an ORR of 36.36%, median PFS and OS of 5.0 and 10.0 m, correspondingly. Subgroup analyses indicated that treatment efficacy closely correlated with patients' Ds‐GPA (Diagnosis‐specified Graded Prognosis Assessment) scores. Mechanistic studies revealed that this regimen likely operates by reducing immune suppression to activate immune function, thereby exerting synergistic anti‐tumor effects. The common AEs include decreased appetite, nausea, leukopenia, hypertension, proteinuria, hand‐foot syndrome, peripheral neuropathy, rash, and thyroid toxicity, most of which are generally mild and can be alleviated with symptomatic treatment.

**Conclusion:**

The combination of nab‐ptx, anlotinib, and PD‐1/L1 inhibitors exhibited substantial efficacy and acceptable safety in the treatment of BM from ES‐SCLC. This novel therapeutic approach holds promise for improving the outcomes for patients with this challenging disease. Further studies are needed to validate these findings and investigate the long‐term benefits of this combination regimen.

## 1 Introduction

Small cell lung cancer (SCLC) is characterized by high aggressiveness, rapid progression, and early dissemination, accounting for about 13%–15% of all lung cancer cases. Approximately two‐thirds of SCLC cases would progress to extensive‐stage SCLC (ES‐SCLC), marked by widespread metastases, often involving brain. In fact, up to 10%–15% of patients with ES‐SCLC are diagnosed with synchronous brain metastases at the initial presentation, while an even higher proportion may develop brain metastases as their disease progress [1]. Brain metastases resulted in substantial morbidity and mortality, and significantly worsen the prognosis, with a median survival time of only 4–6 months. Although the whole‐brain radiation therapy (WBRT), stereotactic radiosurgery (SRS), and systemic chemotherapy have been applied to treat brain metastases, the outcomes are insufficient, necessitating the exploration of novel therapeutic approaches to improve patient prognosis and quality of life [2, 3].

The development of brain metastases in SCLC is attributed to the unique biological characteristics of the disease, including high proliferative capacity, neurotrophic properties, and genetic instability [4]. Conventional chemotherapy agents faces difficulties in achieving adequate concentrations within the central nervous system due to the presence of the blood–brain barrier, thereby impeding their effectiveness against brain metastases. In this context, nab‐paclitaxel (nab‐ptx), a nanoparticle albumin‐bound formulation of paclitaxel, has demonstrated enhanced penetration capacity across the blood–brain barrier, offering a potential advantage in the treatment of SLCL patients with brain metastases [5, 6, 7, 8]. Furthermore, synergistic control of intracranial disease was observed when combining nab‐ptx with anlotinib which is a multi‐targeted small molecular inhibitor, with anti‐angiogenic and anti‐tumor properties [9, 10]. Additionally, PD‐1/L1 inhibitors show potential in activating the host immune system to fight against brain metastases and prevent disease progression [11, 12, 13]. Given the complementary mechanisms of action and the potential synergistic effects of these agents, the combination of nab‐ptx, anlotinib, and PD‐1/L1 inhibitors presents compelling rationale for managing ES‐SCLC patients with brain metastases, warranting thorough investigation. Therefore, this study conducted a retrospective analysis of the efficacy and safety of nab‐ptx, anlotinib, and PD‐1/L1 inhibitors in treating ES‐SCLC with brain metastasis, aiming to explore new approaches to improve the efficacy of treatment for such special population.

## 2 Methods

### 2.1 Study Design

This was a retrospective, single‐center study involving ES‐SCLC patients with brain metastases. The study was performed according with the Declaration of Helsinki and Good Clinical Practice guidelines. Since, this is a retrospective study, the informed consent was waived and the research proposal will be evaluated and approved by the Institutional Review Board or Ethics Committee at Hubei Cancer Hospital (approval no. HBCHEC2021159).

### 2.2 Patient Population

Patients aged 18 years or older who had been diagnosed with histologically ES‐SCLC and radiographically documented brain metastases are enrolled. Patients must have adequate organ function and physical activity to endure the proposed treatment regimen.

### 2.3 Treatment Regimen

Patients received a combination treatment. nab‐ptx was administered intravenously, anlotinib was taken orally, and PD‐1/L1 inhibitors was given as per standard dosing and administration schedules. The treatment cycle, dose modifications, and concomitant medications were managed according to the protocol guidelines.

### 2.4 Clinical Assessments

Comprehensive baseline, including medical history, physical examination, neurological assessment, laboratory tests, imaging studies, and quality‐of‐life assessments will be evaluated. Tumor response will be assessed using radiographic imaging techniques such as magnetic resonance imaging (MRI) or computed tomography (CT) scans according to the Response Evaluation Criteria in Solid Tumors (RECIST) criteria. Progression‐free survival and overall survival will be calculated from the initiation of treatment until disease progression or death, and will be compared with historical controls and existing treatment modalities. Adverse events (AEs) will be graded according to the Common Terminology Criteria for Adverse Events (CTCAE) and closely monitored throughout the treatment period.

### 2.5 Statistical Analysis

Descriptive statistics will be used to summarize patient demographics, treatment outcomes, and safety profiles. Survival analyses will be performed using Kaplan–Meier methods, and subgroup analyses will be conducted to identify potential predictors of treatment response and survival outcomes.

## 3 Results

### 3.1 Patient Characteristics

About 55 ES‐SCLC patients with brain metastases were enrolled in the study. The median age of the patient population was 64 years (range: 47–75 years). The male‐to‐female ratio was 4:1. Most patients had gained 0 or 2 in the Eastern Cooperative Oncology Group (ECOG) performance status. All patients had received prior systemic therapy for SCLC. 40% of patients had a time interval of less than 90 days since the last chemotherapy (CFI), while the rest of them had not received chemotherapy for more than 90 days (Table 1).

**FIGURE 1:**
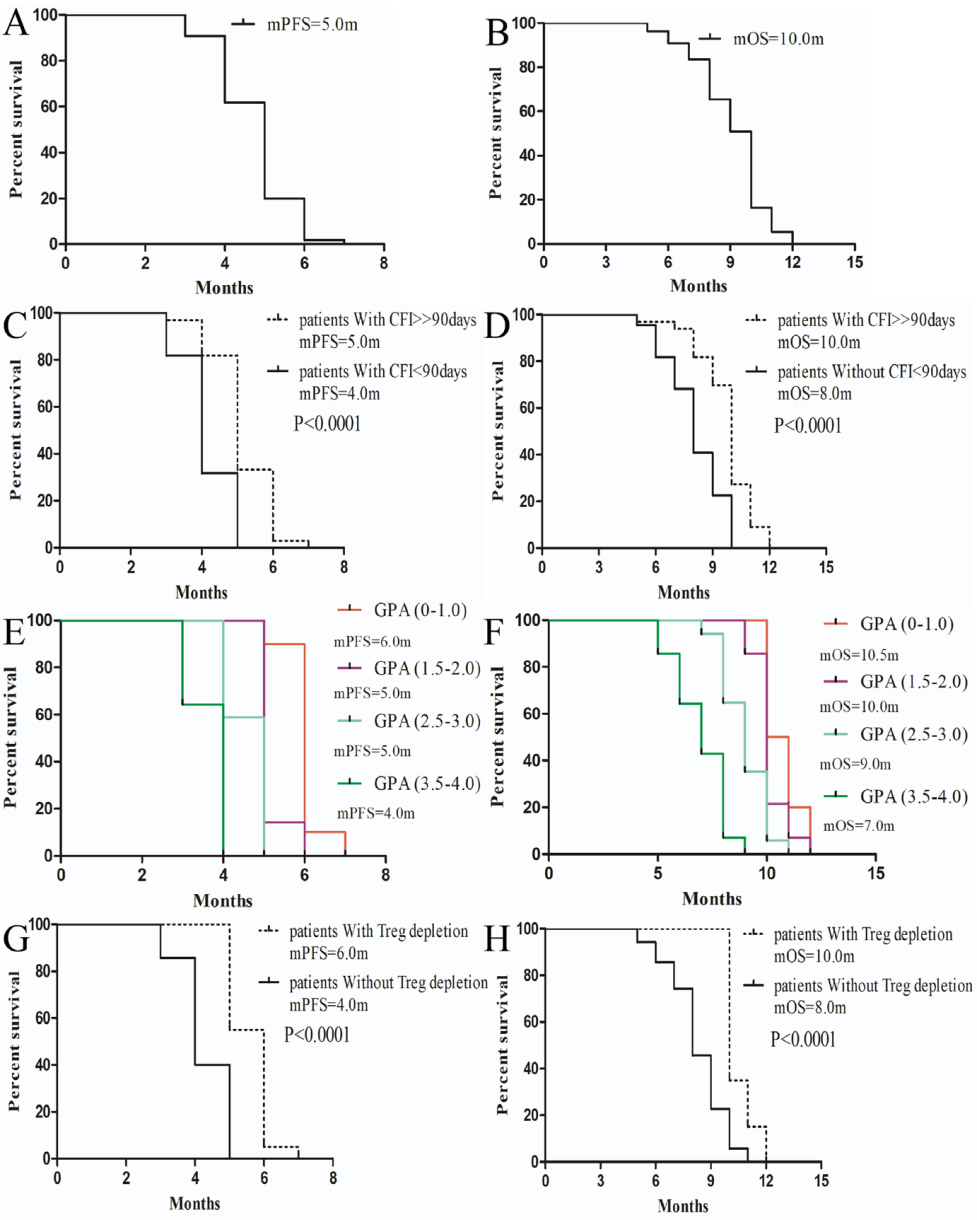
PFS and OS analysis of general population and subgroup patients of ES‐SCLC with BM who accepted the triple‐drug combination of anlotinib, nab‐ptx, and PD‐1/L1 inhibitors in this study. (A, B) The overall PFS and OS in this study. (C, D) Comparisons of PFS and OS between patients with different chemotherapy‐free interval (CFI ≥ 90 days vs. CFI < 90 days). (E, F) Comparisons of PFS and OS among these patients according to the category of different dsGPA score (GPA score (0–1.0), GPA score (1.5–2.0), GPA score (2.5–3.0), and GPA score (3.5–4.0)). (G, H) Comparisons of PFS and OS between these patients with Treg depletion and without Treg depletion (Treg ratio decrease vs. Treg ratio unchanged). CFI, chemotherapy‐free interval (refers to the time from the end of the last chemotherapy session to the start of regimen treatment); Ds‐GPA, diagnosis‐specified Graded Prognosis Assessment; IO, immuno‐oncology; NSCLC, non‐small cell lung cancer; mOS, median overall survival; mPFS, median progression‐free survival; PD‐L1, programmed death ligand 1.

### 3.2 Efficacy Outcomes

All enrolled patients had received at least two cycles of above treatment. Our preliminary results demonstrated that the combination therapy of nab‐ptx, anlotinib, and PD‐1/L1 inhibitors had promising antitumor activity, with an ORR of 36.36% based on radiographic imaging assessments. Impressively, there were 0% of CR and 32.73% of PR indicating substantial tumor regression (Table 2). The median PFS and OS was 5.0 months (95% CI: 3.93–5.56) and 10.0 months (95% CI: 7.47–10.71), respectively. In addition, the regimen demonstrated efficacy in both sensitive‐treatment patients (defined as CFI ≥ 90 days) and refractory‐treatment patients (defined as CFI < 90 days), with ES‐SCLC showing better outcomes in the sensitive‐treatment group. Specifically, patients in the sensitive‐treatment group had a mPFS for 5.0 months compared to 4.0 months in refractory‐treatment group (*p* < 0.0001, HR = 6.896, 95% CI: 2.85–16.68). Additionally, mOS was 10.0 months in the sensitive‐treatment group versus 8.0 months in the refractory‐treatment group (*p* < 0.0001, HR = 0.80, 95% CI: 0.22–1.38). The intracranial response rate was 25% in patients with measurable brain lesions. These findings underscore the efficacy of the combination therapy in controlling and reducing brain metastases, a critical aspect of the disease burden in SCLC (Figure 1).

**TABLE 1:** Baseline clinical characteristics of the study cohort.

Characteristics	No. of patients (%)
Age	
Years	64
Range	47–75
Gender	
Male	42 (76.36%)
Female	13 (23.64%)
Smoking history	
Never smoker	14 (24.44%)
Former smoker	41 (74.55%)
ECOG score	
0–1	44 (80.00%)
≥ 2	11 (20.00%)
Previous radiotherapy	
Yes	18 (32.73%)
No	37 (67.27%)
Median chemotherapy‐free interval	
< 90 days	22 (40.00%)
≥ 90 days	33 (60.00%)
GPA score	
0–1	10 (18.18%)
1.5–2.0	14 (25.45%)
2.5–3.0	17 (30.91%)
3.5–4.0	14 (25.45%)
Brain metastasis	
Measurable	11 (20.00%)
Unmeasurable	44 (80.00%)
Liver metastasis	
Yes	9 (16.36%)
No	46 (83.64%)
Stage	
Limited	9 (16.36%)
Extensive	46 (83.64%)

### 3.3 Subgroup Analyses

In addition to evaluating the overall population, we had screened for subgroups that could benefit most from the regimen. For patients with a pre‐treatment dsGPA score of 0–1.0, 1.5–2.0, 2.5–3.0, and 3.5–4.0, the median progression‐free survival (mPFS) was 6.0, 5.0, 5.0, and 4.0 months, respectively, while the median overall survival (mOS) was 10.5, 10.0, 9.0, and 7.0 months, respectively. The results revealed that the efficacy of this regimen is negatively associated with the patients' pre‐treatment dsGPA score, with lower scores correlating with better efficacy. Therefore, the dsGPA score can be unitized to screen the priority population (Figure 1).

**TABLE 2:** Clinical activity of anlotinib, nab‐ptx, and PD‐1/L1 inhibitors in ES‐SCLC with brain metastasis.

	All patients (*n* = 55)	Chemotherapy‐free interval < 90 days (*n* = 22)	Chemotherapy‐free interval > 90 days (*n* = 33)
Complete response	0	0	0
Partial response	36.36% (20/55)	27.27% (6/22)	42.42% (14/33)
Stable response	30.91% (17/55)	31.82% (7/22)	30.30% (10/33)
Progressive disease	32.73% (18/45)	40.91% (9/22)	27.27% (9/33)
Objective response	36.36%	27.27%	42.42%
Median PFS	5.0 m	4.0 m	5.0 m
Disease control rate	67.27%	59.09%	72.73%
Median OS	10.0 m	8.0 m	10.0 m

### 3.4 Adverse Events

The adverse events were summarized in Table 3. Most of the adverse events are grades 1 and 2. The most common treatment‐related adverse events included loss of appetite (53.33%), nausea (44.44%), vomit (40.00%), fatigue (37.78%), hypertension (31.11%), hand‐foot syndrome (33.33%), proteinuria (26.67%), rash (28.89%), hyperthyroidism (33.33%), and hypothyroidism (35.56%). Less than 20% of patients experienced Grade 3 or above hematological adverse events, with leukopenia and neutropenia being the most frequent serious adverse event, occurring in 7% of patients. Approximately 20% of patients required dose reductions or treatment interruptions due to treatment‐related toxicities. After appropriate management strategies most of these patients were able to resume treatment, highlighting the feasibility of long‐term administration of the combination therapy.

**TABLE 3:** Adverse events of anlotinib, nab‐ptx, and PD‐1/L1 inhibitors in ES‐SCLC with brain metastasis.

Adverse event	Anlotinib, nab‐ptx, and PD‐1/L1 [*n* (%)]	
	Any grade	Grade 3 or 4
Hematological		
Leukopenia	15 (33.33%)	3 (6.67%)
Neutropenia	14 (31.11%)	3 (6.67%)
Thrombocytopenia	12 (26.67%)	2 (4.44%)
Anemia	9 (20.00%)	1 (2.22%)
Nonhematologic		
Decreased appetite	24 (53.33%)	0%
Nausea	20 (44.44%)	0%
Vomit	18 (40.00%)	0%
Fatigue	17 (37.78%)	0%
Hypertension	14 (31.11%)	4 (8.89%)
Hand‐foot syndrome	15 (33.33%)	4 (8.89%)
Proteinuria	12 (26.67%)	4 (8.89%)
Elevated transaminase	10 (22.22%)	4 (8.89%)
Oral ulcer	9 (20.00%)	0%
Stomatitis	10 (22.22%)	0%
Abdominal pain	8 (17.78%)	0%
Diarrhea	8 (17.78%)	0%
Hyperbilirubinemia	6 (13.33%)	0%
Elevated LDH	8 (17.78%)	0%
ALP increased	7 (15.56%)	0%
Elevated GGT	5 (11.11%)	0%
Hypoproteinemia	6 (13.33%)	0%
Dysphagia	5 (11.11%)	0%
Dysphonia	6 (13.33%)	0%
Bleeding	0%	0%
Immunological		
Hypothyroidism	16 (35.56%)	0%
Hyperthyroidism	15 (33.33%)	0%
Rash	13 (28.89%)	4 (8.89%)
Hepatitis	8 (17.78%)	3 (6.67%)
Itching	9 (20.00%)	3 (6.68%)
Pneumonia	5 (11.11%)	3 (6.67%)
Infusion reaction	4 (8.89%)	1 (2.22%)
Nephritis	3 (6.66%)	1 (2.22%)

### 3.5 Mechanistic Exploration

Considering the efficacy of this regimen is associated with immune activation, we compared the peripheral blood lymphocyte proportions between pre‐ and post‐treatment. Preliminary results suggest that Treg depletion contributed to the treatment efficacy. Patients with Treg depletion after treatment exhibit significantly higher efficacy compared to those without Treg depletion (mPFS 6.0 m vs. 4.0 m, *p* < 0.0001, HR = 9.770, 95% CI: 4.27–22.38; mOS 10.0 m vs. 8.0 m, *p* < 0.0001, HR = 6.41, 95% CI: 3.10–13.27). Therefore, reducing immune suppression and further activating the immune system resulted in synergistic anti‐cancer effects by the regimen.

## 4 Discussion

Small cell lung cancer (SCLC) patients with brain metastases have been faced with insufficient treatment options. Radiotherapy is the mainstream treatment for brain metastases, but it only provides short‐term efficacy, and may result in adverse effects such as cognitive impairment and bone marrow suppression [1]. While immunotherapy has brought long‐term survival benefits to some patients with ES‐SCLC [14, 15], only a minority effective [16], with even fewer in those with brain metastases. Therefore, it is of significance to explore new approaches for treating SCLC patients with brain metastases [17].

Considering that combination therapy has become the trend in treatment [18, 19, 20], and with an increasing amount of data on multi‐drug combinations in the treatment of NSCLC with brain metastases, there is some rationale for employing multi‐drug combinations in the treatment of SCLC with brain metastases [21, 22]. The pharmacological treatment not only provides a valuable complement to the treatment options for SCLC with brain metastases but also poses a certain challenge to the role of radiotherapy [23, 24]. Although the number and types of drugs available for SCLC are limited compared to NSCLC, a combination of chemotherapy, immune checkpoint blockade, and anti‐angiogenesis therapy is likely to be a reasonable choice [10, 25]. However, further optimization and exploration are needed in terms of specific drug combinations. As the efficacy and safety of various immunotherapeutic drugs in treating SCLC is similar in spite of no comparative data, the selection of immunotherapeutic drugs may not have a significant impact in this setting [15, 18, 20, 26, 27, 28, 29]. In terms of anti‐angiogenesis therapy, anlotinib, as a small molecule inhibitor targeting VEGF, has significant advantages [9]. Regarding chemotherapy drugs, albumin‐bound paclitaxel, with its superior penetration compared to paclitaxel [5, 6] and its potential for combination with immunotherapy, has become a optimal choice for this regimen [19, 30]. Our preliminary research results suggest that combination therapy can significantly improve the efficacy for patients with ES‐SCLC and brain metastases, as evidenced by the ORR of 36%, significantly surpassing historical benchmarks [31, 32]. Furthermore, the median PFS of 5 months and OS of 10 months represent substantial improvements compared to standard treatments and previous research reports [9, 32, 33], indicating meaningful clinical benefits for patients. Notably, the high intracranial response rate of 25% for measured lesion and prolonged duration of intracranial response highlight the efficacy of the combination therapy in controlling and reducing brain metastases, addressing a critical aspect of the disease burden in ES‐SCLC. In addition to significant therapeutic effects, our research has also, for the first time, identified that the scoring of dsGPA [34] can be applied to predict the efficacy of treatment in patients suffering from brain metastases. Lower scores are associated with better efficacy [34]. Furthermore, through comparing the proportions of immune cells in peripheral blood pre‐ and post‐treatment, we found that patients with a decrease in Treg cell proportion (Treg depletion) had significantly higher efficacy compared to those with unchanged Treg proportions [19]. This suggests that the downregulation of Treg proportions not only serves as a potential biomarker for identifying patient populations likely to benefit from this regimen but also implies that the regimen's mechanism of action may involve reducing immune suppression. This, in turn, could enhance immune system activation and promote synergistic anti‐tumor effects [35].

As the dosage of albumin‐bound paclitaxel used in this study is relatively low [19, 36], and the treatment regimen does not include platinum‐based drugs [11, 30], there was no cumulative occurrence of toxic side effects by the triple‐drug combination [35, 37, 38]. These data not only highlight the potential of the triple‐drug combination in managing ES‐SCLC patients with brain metastasis but also offer valuable insights and references for future research in this area. Nevertheless, due to the complexity of multiple drug combinations and the intricate composition of the immune microenvironment, several challenges remain with this regimen. These include the optimization of combination therapy patterns, elucidation of the mechanism of action [29, 39], and the need for further investigation into predicting efficacy biomarkers [40] and their discrimination [41]. Addressing these issues is a central focus for our ongoing research.

It is important to acknowledge certain drawbacks of the study, including its retrospective property, small sample size, and the need for confirmation in larger, multicenter cohorts. Additionally, the optimal sequencing of the combination therapy with other treatment options, such as radiotherapy [24, 42] or immunotherapy [43], warrants further investigation to maximize therapeutic synergy and minimize toxicities. Furthermore, ongoing efforts to identify additional predictive biomarkers [40, 44, 45] and understand the interplay of the tumor microenvironment [46, 47] with the combination therapy will be crucial for advancing precision medicine approaches in ES‐SCLC with brain metastases.

## 5 Conclusion

To the best of our knowledge, this is the first retrospective study for evaluating the clinical efficacy and tolerability of the combination of nab‐ptx, anlotinib, and PD‐1/L1 inhibitors in treatment of ES‐SCLC patients with brain metastases in. Our results demonstrated that the triple combination is efficacious and tolerable in this population. The robust clinical outcomes and mechanistic understanding generated from this study have significant implications for the treatment of ES‐SCLC with brain metastases. Further studies are needed to validate these findings and investigate the long‐term benefits of this combination regimen.

## Author Contributions

**Xiaobing Li:** writing – original draft (lead). **De Wu:** methodology (lead). **Yi Peng:** data curation (lead). **Jing Tang:** formal analysis (lead). **Yuebing Wu:** conceptualization (lead).

## Ethics Statement

The authors are responsible for the accuracy and integrity of all aspects of the study and are committed to addressing any questions regarding its validity. The study was carried out according to the guidelines outlined in the Declaration of Helsinki (revised in 2013). The retrospective trial received approval from the Ethics Committee of Hubei Cancer Hospital, Tongji Medical College, Tongji Medical College, Huazhong University of Science and Technology (Wuhan, China) (No. HBCHEC2021159), Since this is a retrospective study, the informed consent was waived.

## Conflicts of Interest

The authors declare no conflicts of interest.

## Data Availability

The data involved in this study are available upon reasonable request from the corresponding author.
